# Seeking Goldilocks During Evolution of Drug Resistance

**DOI:** 10.1371/journal.pbio.2001872

**Published:** 2017-02-03

**Authors:** Gavin Sherlock, Dmitri A. Petrov

**Affiliations:** 1 Department of Genetics, Stanford University, Stanford, California, United States of America; 2 Department of Biology, Stanford University, Stanford, California, United States of America

## Abstract

Speciation can occur when a population is split and the resulting subpopulations evolve independently, accumulating mutations over time that make them incompatible with one another. It is thought that such incompatible mutations, known as Bateson–Dobzhansky–Muller (BDM) incompatibilities, may arise when the two populations face different environments, which impose different selective pressures. However, a new study in *PLOS Biology* by Ono et al. finds that the first-step mutations selected in yeast populations evolving in parallel in the presence of the antifungal drug nystatin are frequently incompatible with one another. This incompatibility is environment dependent, such that the combination of two incompatible alleles can become advantageous under increasing drug concentrations. This suggests that the activity for the affected pathway must have an optimum level, the value of which varies according to the drug concentration. It is likely that many biological processes similarly have an optimum under a given environment and many single-step adaptive ways to reach it; thus, not only should BDM incompatibilities commonly arise during parallel evolution, they might be virtually inevitable, as the combination of two such steps is likely to overshoot the optimum.

## Introduction

Genetic incompatibilities arise due to **epistasis**, a term coined by Bateson [1] to describe how the action of one gene could be “stopped” by another. While epistasis is still used in this manner, more generally, it means that the effect of an allele of one gene depends on the genetic background upon which it is found. Dobzhansky and Muller further speculated that in independently evolving populations, the accumulation of such interactions could lay the basis for their emergence as two species [2, 3]. In the simplest case of a Bateson–Dobzhansky–Muller (BDM) incompatibility, alleles of two genes interact such that either alone may be neutral or even beneficial, but in combination they are deleterious, decreasing the fitness of an individual carrying them both [4, 5]. The presence of BDM incompatibilities would be expected to diminish gene flow between populations carrying such alleles, as it would decrease the fitness of hybrids generated by parents from the two populations. Deducing which genic incompatibilities resulted in speciation between closely related taxa can be challenging; although, in some cases, BDM alleles have been uncovered, for example, in flies [6, 7], wasps [8], and yeast [9]. Even then, it is almost impossible to tell whether these changes caused speciation to occur or arose after species boundaries were established. A major challenge facing evolutionary geneticists is to understand in more than a handful of ad hoc cases the root causes of epistatic interactions that may lead to new species.

What is the likelihood that any two mutations result in a BDM pair? And, particularly, how often do adaptive mutations form BDM pairs? The answers to these questions would shed light on whether speciation is likelier to occur when populations experience divergent selective pressures as they adapt to different local environments (ecological speciation), or whether BDM pairs can arise when population(s) adapt to the same environment. A powerful approach to answering these questions is microbial experimental evolution, which is a prospective rather than retrospective endeavor. Experimental evolution has many advantages, including short generation times for the organisms under study (meaning that they can be evolved for many generations), precise control over the experimental conditions (the “ecological theater” in which the “evolutionary plays” are enacted [10]), the ability to specify the population size, and, perhaps most importantly, the ability to freeze down populations at periodic intervals that can be later revived for further study, preserving a frozen yet living fossil record of the experiment (see [11]). Furthermore, because microbes have small genomes, it is cheap and straightforward to identify by high throughput sequencing the beneficial mutations in dozens or even hundreds of clones of interest (e.g., [12–14]) or even in entire populations [15–17]. Finally, these relatively simple experiments can be performed in many parallel replicates, allowing for the tape of life to be played multiple times simultaneously [18].

## Adaptive Evolution and Epistasis

The scenario of ecological speciation, whereby populations become isolated due to divergent selection, can be reproduced using experimental evolution—Dettman and colleagues evolved yeast populations under different experimental conditions [19] either in the presence of high salt or low glucose. When crossing individuals from a population adapted to one environment with individuals from a population adapted to the other environment, they found that environment-specific adaptations had resulted in postzygotic isolation, wherein the hybrids suffered from a reduced rate of mitotic reproduction and reduced efficiency of meiotic reproduction. By contrast, hybrids between parallel lines that were evolved under the same conditions did not show reduced fitness, although the hybrids had some decrease in meiotic efficiency compared to that of pure populations. A subsequent study characterized epistasis between beneficial alleles that arose under a single selective pressure and found that while there was pervasive negative epistasis between adaptive mutations (the whole being less than the sum of the parts because of diminishing returns), only in one specific case were beneficial alleles actually incompatible [20]. In that case, the two mutations, in *MTH1* and *HXT6/7*, were likely to affect the same pathway and in the same direction, suggesting the existence of a fitness optimum for that pathway under the evolutionary conditions and that the double mutant overshot it. While several studies have found abundant evidence for diminishing returns epistasis for adaptive mutations [21–24], likely due to the incremental fitness of beneficial alleles being inversely proportional to the fitness of the individual in which they occur [25], there are few examples of beneficial alleles being incompatible. However, theoretical considerations [26, 27] suggest that should strongly adaptive mutations be sufficiently likely, the first mutation that rises to very high frequency may be fairly close to the fitness optimum. Consequently, the combination of two early-arising highly beneficial alleles is likely to overshoot the fitness optimum. Furthermore, in the case of an adapting diploid, highly beneficial alleles are likely to be overdominant, i.e., show heterozygote advantage [27]—a prediction for which there is some limited empirical support [28]. In the case of beneficial mutations occurring at different loci, this might lead to mutually exclusive mutations—essentially BDM incompatibilities.

## Fitness, Epistasis, and the Environment

As an organism adapts to one environment, it is likely inevitable that there will be evolutionary trade-offs, as a given adaptive allele may affect other traits, a phenomenon known as pleiotropy. Indeed, beneficial mutants isolated from experimental evolution studies can have reduced fitness in alternative environments, although not universally so (e.g., [29–31]). Importantly, epistatic interactions between beneficial alleles can be altered as a function of the environment; for example, Flynn and colleagues [29] examined the fitness of all possible combinations of the first five beneficial mutations in a laboratory-evolved population of *Escherichia coli* in two different environments—they found that in the two environments, mutations tended to remain beneficial, and the overall pattern of epistasis was of diminishing returns. However, there were also some environment-dependent features: several mutations were detrimental on specific genetic backgrounds, indicating the presence of antagonistic interactions that were absent in the environment in which they were selected. Clearly, understanding adaptation and genetic incompatibilities requires consideration of the combined effects and interactions between epistasis and pleiotropy across different environments.

**Fig 1:**
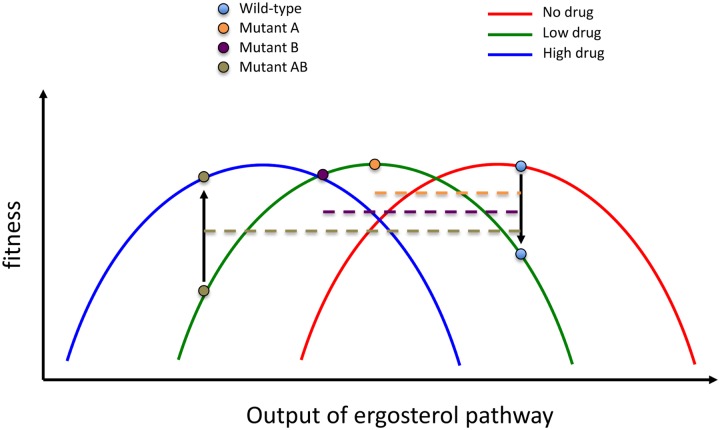
A shifting optimum under different environmental conditions. The wild-type genotype has high fitness in the absence of the drug, but the presence of the drug shifts the optimum to the left, and the wild-type fitness declines. Mutants A and B are both more fit in the presence of the low drug concentration, but when combined, the AB double mutant overshoots the fitness optimum to have lower fitness even than the wild type. Increasing the drug concentration shifts the optimum further to the left, and now the double mutant has high fitness.

In this issue of *PLOS Biology*, Ono and colleagues [32] describe work in which they systematically tested the pairwise epistasis between first-step adaptive mutations that were selected when *Saccharomyces cerevisiae* was evolved in the presence of the antifungal nystatin [33]. Strikingly, Ono et al. [32] found that in as many as a third of these pairwise combinations, the double mutant was less fit than either of the two single mutants, a phenomenon known as reciprocal sign epistasis that can result in a BDM incompatibility. All these mutations are in genes that function in the ergosterol biosynthesis pathway—many, though not all, of the mutations are likely to be loss-of-function mutations. Nystatin acts by binding ergosterol in the cell membrane and, when present at a sufficient concentration, alters membrane permeability and stability, which can lead to K^+^ leakage and cell death [34, 35]. Producing **less** ergosterol is therefore beneficial in the presence of nystatin. However, because ergosterol is an essential membrane constituent, there should be a “Goldilocks zone” for ergosterol biosynthesis, and while the single mutants land quite near it, the double mutants frequently overshoot it (Fig 1). Ono et al. tested for this possibility by assaying fitness of these same pairwise genetic combinations in the presence of an **increased** amount of nystatin; they found that these double mutant combinations now became beneficial, suggesting that the Goldilocks zone had moved.

It is an exciting time to be using experimental evolution to gain insight into the functioning of biological systems, including the mechanics of the evolutionary process. For a century, biologists have used genetic screens to identify mutants that have lost the ability to perform a given function and used said mutants to reconstruct pathways. The ability now to select beneficial alleles that improve cellular fitness, in parallel (within the same culture or in replicate cultures) under many different experimental conditions, and to inexpensively identify the molecular nature of these mutations now allows us to conduct **adaptive genetics**, in which we can systematically examine the nature, phenotypic consequences, and trade-offs of beneficial mutations. Furthermore, technological developments such as high throughput sequencing and molecular tagging of clones (DNA barcoding [36, 37]) allow the straightforward identification of adaptive clones and measurement of their fitness precisely, cheaply, and in a highly parallel fashion [38]. Adaptive mutants arising under one evolutionary condition can be isolated and their fitness remeasured under other conditions to generate a joint distribution of fitness effects. This procedure can be reiterated under as many conditions as logistically feasible, opening up an unprecedented view of the consequences of beneficial mutations. Going forward, one major challenge will be to combine beneficial alleles and generate a global view of epistasis, specifically how the intensity and sign of epistasis for any pair of alleles change as a function of the environment. Another major challenge will be to determine whether different types of epistasis will be prevalent for similar versus dissimilar environments that were used to select the adaptive mutations—i.e., how local is adaptation? Dual barcoding systems (e.g., [39]) may hold some promise in regard to measuring epistasis on a large scale, although challenges remain in how to systematically measure the fitness of thousands of pairwise combinations of beneficial alleles under dozens of conditions. The data generated once these challenges are met will produce an embarrassment of riches that will enable us to acquire a deeper understanding of speciation and the evolutionary process.
